# Gene Expression Profile Analysis of Type 2 Diabetic Mouse Liver

**DOI:** 10.1371/journal.pone.0057766

**Published:** 2013-03-01

**Authors:** Fang Zhang, Xiang Xu, Yi Zhang, Ben Zhou, Zhishui He, Qiwei Zhai

**Affiliations:** Key Laboratory of Nutrition and Metabolism, Institute for Nutritional Sciences, Shanghai Institutes for Biological Sciences, Chinese Academy of Sciences, Shanghai, China; Rutgers University, United States of America

## Abstract

Liver plays a key role in glucose metabolism and homeostasis, and impaired hepatic glucose metabolism contributes to the development of type 2 diabetes. However, the precise gene expression profile of diabetic liver and its association with diabetes and related diseases are yet to be further elucidated. In this study, we detected the gene expression profile by high-throughput sequencing in 9-week-old normal and type 2 diabetic db/db mouse liver. Totally 12132 genes were detected, and 2627 genes were significantly changed in diabetic mouse liver. Biological process analysis showed that the upregulated genes in diabetic mouse liver were mainly enriched in metabolic processes. Surprisingly, the downregulated genes in diabetic mouse liver were mainly enriched in immune-related processes, although all the altered genes were still mainly enriched in metabolic processes. Similarly, KEGG pathway analysis showed that metabolic pathways were the major pathways altered in diabetic mouse liver, and downregulated genes were enriched in immune and cancer pathways. Analysis of the key enzyme genes in fatty acid and glucose metabolism showed that some key enzyme genes were significantly increased and none of the detected key enzyme genes were decreased. In addition, FunDo analysis showed that liver cancer and hepatitis were most likely to be associated with diabetes. Taken together, this study provides the digital gene expression profile of diabetic mouse liver, and demonstrates the main diabetes-associated hepatic biological processes, pathways, key enzyme genes in fatty acid and glucose metabolism and potential hepatic diseases.

## Introduction

Type 2 diabetes has become epidemic in both developed countries and developing countries with a rapid change of lifestyle [1], [2]. The predominant features of type 2 diabetes include increased fasting blood glucose levels, as well as disturbed peripheral glucose utilization. The prevalence of type 2 diabetes and the limitations of currently available preventative and therapeutic options highlight the need for a more complete understanding of the pathogenesis of type 2 diabetes [3].

Liver has a key role in glucose metabolism and homeostasis. Hepatic glucose metabolism mainly includes glucose transport, glycolysis, gluconeogenesis, glycogen synthesis and glycogenolysis [4], and impaired hepatic glucose metabolism will lead to the development of type 2 diabetes [4], [5]. Many key enzymes involved in hepatic glucose metabolism, such as glycogen phosphorylase (GP, *Pygl*) the rate-limiting enzymes of glycogenolysis [6], fructose-1,6-bisphosphatase (FBPase, *Fbp1*) and glucose 6-phosphatase (G6pase, *G6pc*) the major control points in the pathway of gluconeogenesis [7], [8], and glucokinase (GK/HK, *Gck*) the key enzyme of glycolysis [9], are the potential targets for diabetes. Meanwhile, hepatic transcriptional factors, such as Pparα and Hnf4α, are well-known to regulate glucose homeostasis, and involved in the development of hyperglycemia [10], [11]. Impaired hepatic fatty acid metabolism, including fatty acid oxidation, synthesis and storage, is also involved in the development of type 2 diabetes [12], [13], [14]. However, an overview of the hepatic glucose and fatty acid metabolism in diabetic subjects needs to be further investigated.

Microarray technology has been applied to study the genome-wide gene expression levels in type 1 diabetic tissues, such as rat muscle and mouse liver [15], [16]. Microarray technology has also been used in type 2 diabetic studies to reveal the gene expression profiles in insulin-sensitive tissues from pre-diabetic and diabetic Zucker and Goto-kakizaki rats [17], [18]. Similarly, mouse hepatic gene expression profiles by microarray analysis in diet-induced obesity and diabetes reveal a transcriptional adaptation to long-term high-fat diet, and provided potential molecular mechanisms underlying the development and maintenance of obesity and diabetes [19]. Microarray experiments using type 2 diabetic mouse model also suggest a role of hepatic lipogenic capacity in diabetes susceptibility [20]. Recently, high-throughput sequencing, as an attractive alternative to microarrays for transcriptome profiling, shows major advances in robustness, resolution and reproducibility based on its relatively unbiased and direct digital readout with sequencing by synthesis method [21], . In terms of both profiling coverage and quantitative accuracy of high-throughput sequencing, the application of this new technology in transcriptome profiling of diabetic samples is important to further understand the underlying mechanisms.

In this study, we detected gene expression profile in normal and type 2 diabetic db/db mouse liver by high-throughput sequencing, and showed the main diabetes-associated hepatic biological processes, pathways, key enzyme genes in fatty acid and glucose metabolism and potential hepatic diseases.

## Results

### High-throughput Sequencing and the Top Abundance Change and Fold Change Genes in Normal and Diabetic Mouse Liver

**Figure 1:**
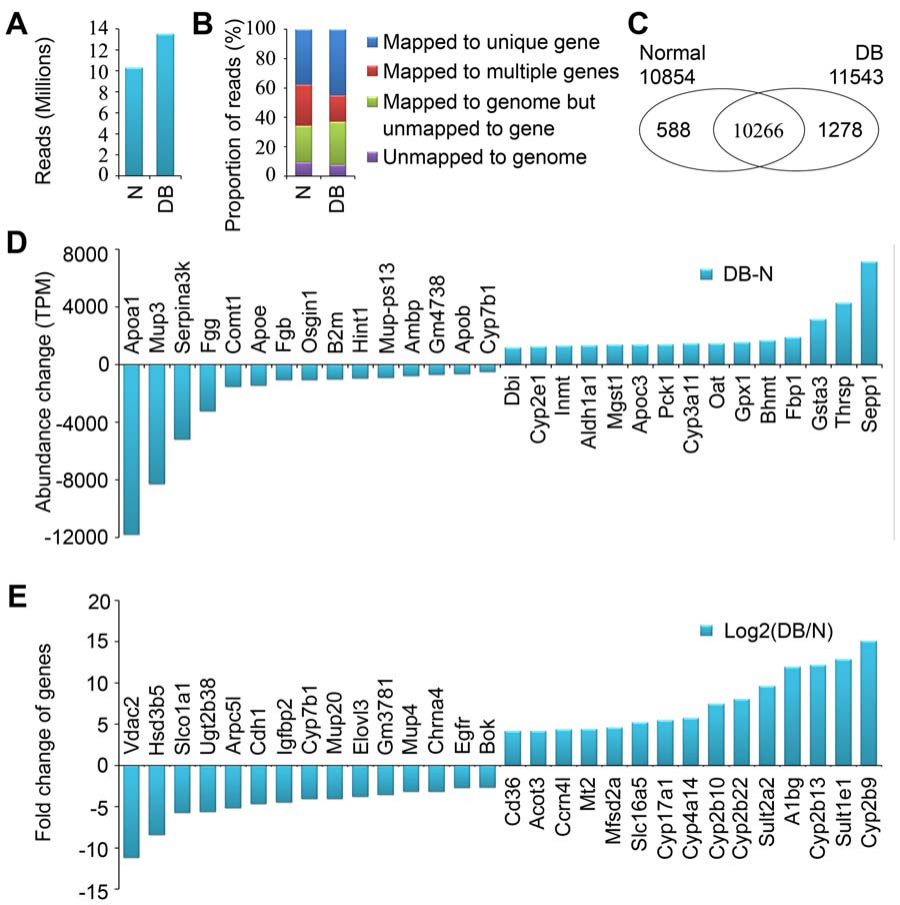
Sequencing and mapping messages of hepatic mRNA profiling of normal and diabetic mice. (A) The high-quality clean reads from high-throughput sequencing. Total liver RNA from 9-week-old normal (N) and diabetic (DB) db/db mice was used to prepare the high-throughput sequencing library. (B) The proportions of high-quality clean reads unmapped and/or mapped to unique genes, multiple genes and genome. (C) The number of genes detected in normal and diabetic mouse liver. (D) The top 15 abundance change genes downregulated or upregulated in diabetic mouse liver. (E) The top 15 fold change genes downregulated or upregulated in diabetic mouse liver.

To survey the gene expression profile in diabetic mouse liver, high-throughput sequencing was applied in this study. Finally, we obtained over 10 and 13 million reads of high quality clean tags from 9-week-old normal and type 2 diabetic db/db mice respectively (Figure 1A). In these high quality clean tags, averagely about 91.4%, 72.9% and 41.4% reads can be mapped to annotated mouse genome, genes, and unique genes respectively (Figure 1B). Totally 12132 unique genes were detected, and 10854 and 11543 unique genes were detected and quantified from normal and diabetic samples respectively, which shared 10266 genes in common (Figure 1C). Based on the high-throughput sequencing data, the top 15 abundance change genes upregulated and downregulated respectively in diabetic mouse liver were shown in Figure 1D. *Apoa1* encoding Apolipoprotein A-I, the major protein component of high density lipoprotein and playing an important role in lipid metabolism, *Mup3* (major urinary protein 3) and *Serpina3k* (serine (or cysteine) peptidase inhibitor, clade A, member 3K) were the top 3 abundance change genes downregulated in diabetic mouse liver (Figure 1D). *Sepp1* (selenoprotein P), *Thrsp* (thyroid hormone responsive SPOT14 homolog) and *Gsta3* (glutathione S-transferase, alpha 3) were the top 3 abundance change genes upregulated (Figure 1D). In addition, we showed the top 15 fold changes of genes upregulated and downregulated respectively in diabetic mouse liver in Figure 1E. Interestingly, *Cyp2b9*, *Cyp2b13*, *Cyp2b22*, *Cyp2b10*, *Cyp4a14* and *Cyp17a1* in the superfamily of Cytochrome P450, which catalyzes the oxidation of organic substances, were in the top 15 fold change genes upregulated in diabetic mouse liver (Figure 1E). *Vdac2* (voltage-dependent anion channel 2), *Hsd3b5* (hydroxy-delta-5-steroid dehydrogenase) and *Slco1a1* (solute carrier organic anion transporter family, member 1a1) were the top 3 fold changes of downregulated genes (Figure 1E).

### Differentially Expressed Genes and Clustering

Among the 10854 and 11543 unique genes detected from normal and diabetic mouse liver samples, the number of differentially expressed genes were quantified and shown in Figure S1A. To avoid the possible noise signal from high-throughput sequencing, the genes with an average transcripts per million (TPM) less than 1 were excluded. The remained 8551 genes and their abundance were shown in Table S1, and were used to calculate the fold changes and false discovery rate (FDR). In this study, the absolute fold change no less than 1.5 and FDR less than 0.001 were used to define the differentially expressed genes. According to this definition, totally 2627 genes were differentially expressed between normal and diabetic mouse liver samples (Figure S1A). To gain insights into the 2627 differentially expressed genes in diabetic mouse liver, we divided them into 2 clusters based on upregulated and downregulated pattern in diabetic mouse liver (Figure S1B). Cluster A and Cluster B included 1933 upregulated and 694 downregulated genes in diabetic mouse liver respectively.

### The Main Biological Processes and Pathways Altered in Diabetic Mouse Liver

**Figure 2:**
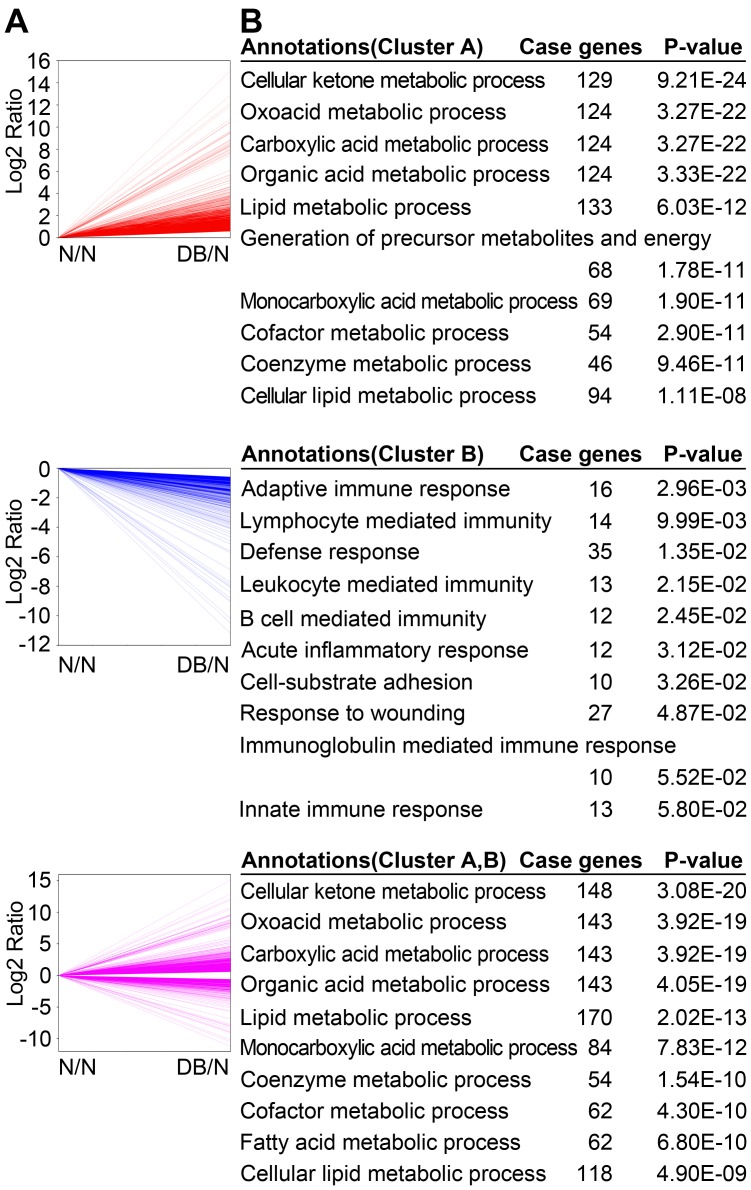
Genes and the related biological processes altered in diabetic mouse liver. (A) The 8551 selected genes as described in Figure S1A were separated into two distinct clusters according to the genes upregulated or downregulated in diabetic (DB) mouse liver compared with normal (N) control. Red lines indicate Cluster A including 1933 upregulated genes in diabetic mouse liver. Blue lines indicate Cluster B including 694 downregulated genes . Purple lines indicate the total 2627 altered genes, which include all genes in Cluster A and B. (B) The clustered genes were assigned to different biological processes based on Gene Ontology using the web tool DAVID. The top 10 biological functions and the case genes in each cluster ranked by *P*-value were listed (case genes ≥ 10).

To investigate the possible biologic functions of the genes affected in diabetic mouse liver, the Cluster A and B with 1933 genes upregulated and 694 genes downregulated respectively were analyzed by Gene Ontology (Figure 2A). Cluster A genes were strongly enriched in cellular ketone and organic acid metabolic process, especially in their subprocess oxoacid metabolic process and its downstream carboxylic acid metabolic process, and even more specifically in monocarboxylic acid (Figure 2B). Moreover, lipid metabolic process and its subprocess cellular lipid metabolic process, and cofactor metabolic process and its subprocess coenzyme metabolic process were enriched in Cluster A (Figure 2B). Surprisingly, Cluster B genes were mainly enriched in immune-related processes, such as adaptive immune response and lymphocyte mediated immunity, especially their downstream process B cell mediated immunity and its subprocess immunoglobulin mediated immune response (Figure 2B). These data implicate that diabetes is associated with immune response. The 2627 differentially expressed genes in diabetic mouse liver including Cluster A and Cluster B mainly enriched in metabolic processes, which is similar with Cluster A (Figure 2B). These data suggest that metabolic processes are the major processes altered in diabetic mouse liver.

Furthermore, KEGG pathway analysis was performed to further elucidate the biological functions of the gene clusters affected in diabetic mouse liver. The top 5 KEGG pathways significantly correlated with type 2 diabetes were shown in Table 1 with associated genes. These pathway analysis data further confirmed that metabolic pathways in liver are the main biological processes altered in diabetic mouse liver. Besides, the downregulated genes were enriched in complement and coagulation cascades and pathway in cancer, suggesting that diabetes is correlated with immunity and cancer.

**Table 1:** The associated genes in the top 5 KEGG pathways significantly altered in diabetic mouse liver.

	KEGG Pathway	
**Cluster A**Upregulated1933 genes	**Drug metabolism**	
	CYP3A25-CYP2B9-UGT2B1-GSTM5-CYP2A12-GSTM3-GSTM4-FMO1-FMO2-GSTK1-FMO3-GSTZ1-UGT2A3-GSTO1-CYP3A44-GSTA3-CYP2C55-CYP3A16-CYP3A13-LOC639023-CYP3A11-MAOB-CYP2C29-GSTT1-CYP2B13-GSTT2-CYP2B10-CYP2C50-MGST3-CYP2A22-UGT2B36-LOC100046051-AOX1-UGT2B5-CYP2A5-CYP3A59-CYP2C38-CYP2C39-MGST1 P = 1.68E-13	
	**Valine, leucine and isoleucine degradation**	
	HSD17B10-ACADSB-EHHADH-ECHS1-ACAT2-ACAT1-ALDH3A2-AUH-MCCC2-IVD-MCEE-ACAD8-HADH-HMGCL-BCKDHA-ALDH6A1-ACAA2-ACADM-ACADS-ALDH7A1-ALDH1B1-DLD-AOX1-ABAT-HIBCH-PCCA P = 5.97E-11	
	**Metabolism of xenobiotics by cytochrome P450**	
	CYP3A25-CYP2B9-UGT2B1-GSTM5-GSTM3-GSTM4-GSTK1-GSTZ1-GSTO1-UGT2A3-CYP3A44-GSTA3-CYP2C55-CYP3A16-CYP3A13-CYP1A1-LOC639023-CYP3A11-CYP2C29-GSTT1-EPHX1-CYP2B13-GSTT2-CYP2B10-CYP2C50-MGST3-UGT2B36-UGT2B5-CYP3A59-CYP2C38-CYP2C39-MGST1 P = 8.61E-11	
	**Retinol metabolism**	
	CYP3A25-CYP2B9-UGT2B1-ALDH1A1-CYP2A12-GM10774-ALDH1A7-UGT2A3-CYP3A44-CYP2C55-CYP3A16-CYP3A13-CYP1A1-LOC639023-CYP3A11-CYP2C29-CYP26A1-CYP2B13-CYP2B10-CYP2C50-CYP2A22-UGT2B36-DHRS4-DGAT1-UGT2B5-CYP2A5-CYP3A59-RDH16-CYP4A14-CYP2C38-CYP2C39-RETSAT P = 1.09E-09	
	**Oxidative phosphorylation**	
	UQCRC2-ATP5D-ATP5E-NDUFB6-UQCRC1-NDUFB9-ATP6AP1-ATP5B-COX7B-UQCRFS1-COX5A-GM10039-UQCRQ-ATP5G3-NDUFS7-ATP6V0E-GM4943-NDUFS8-COX6B1-ATP6V0D1-NDUFS2-ATP5K-NDUFS1-COX15-NDUFA2-NDUFB10-NDUFA9-NDUFA6-NDUFA7-COX4I1-NDUFA10-LHPP-ATP6V1D-NDUFV3-SDHA-ATP6V1C1-GM4459-NDUFV1-ATP6V1E1-ATP5A1-ATP6V0A2 P = 2.53E-08	
**Cluster B**Downregulated694 genes	**Complement and coagulation cascades**	
	F11-KNG2-CFB-C6-SERPING1-C1QC-PLG-C8A-C1QA-C8B-FGG-FGA-FGB-SERPINF2 P = 5.50E-04	
	**Pathways in cancer**	
	HSP90AB1-E2F3-PPARD-CDH1-NFKB2-ITGB1-CTNNB1-ITGAV-HHIP-TPR-PIK3R1-CSF1R-EGFR-BMP4-BMP2-COL4A1-HSP90AA1-RELA-SMAD4-APPL1-STK4-FZD7-FZD6-DAPK1-VEGFB-CBLC-LAMA3-PDGFRA-JAK1 P = 2.13E-03	
**Cluster A and B**2627 genes	**Drug metabolism**	P = 7.44E-12
	**Metabolism of xenobiotics by cytochrome P450**	P = 1.46E-09
	**Retinol metabolism**	P = 2.75E-09
	**Valine, leucine and isoleucine degradation**	P = 2.23E-08
	**Steroid hormone biosynthesis**	P = 2.14E-06

### The Upregulation of Key Enzyme Genes in Fatty Acid and Glucose Metabolism in Diabetic Mouse Liver

**Figure 3:**
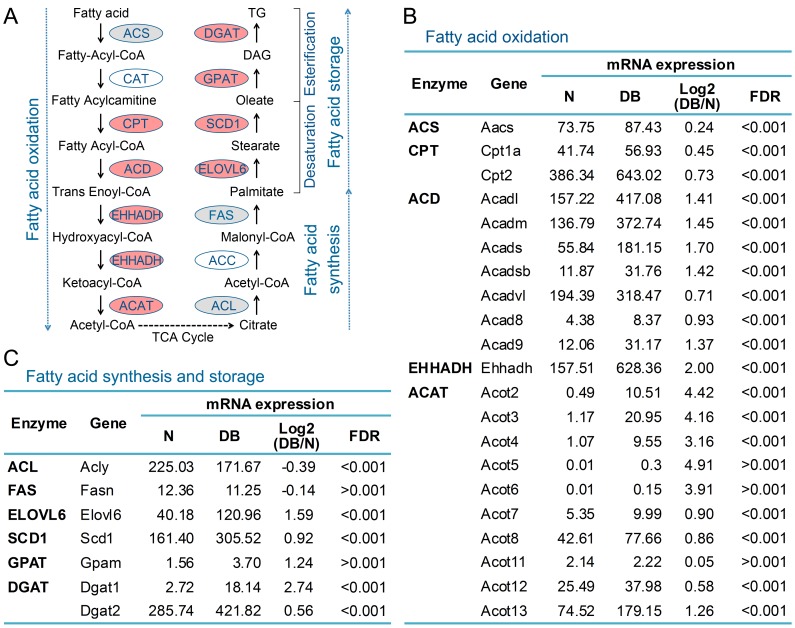
The expression of enzymes directly participated in fatty acid β-oxidation, synthesis and storage was increased or not significantly changed in diabetic mouse liver. (A) Schematic of fatty acid metabolism and the expression of enzymes directly participated in fatty acid oxidation, synthesis and storage in diabetic mouse liver. Red color represents the enzyme genes upregulated in diabetic mouse liver with fold change ≥ 1.5 and FDR < 0.001, gray color indicates no significant change, and white color indicates the genes were not detected. (B–C) The mRNA levels of the enzymes directly participated in fatty acid oxidation, synthesis and storage in normal (N) and diabetic (DB) mouse liver. ACS, acetyl-CoA synthesis; CAT, carnitine acyl transferase; CPT, carnitine palmitoyltransferase; ACD, acyl-CoA dehydrogenase; EHHADH, enoyl-CoA-hydratase/3-hydroxyacyl-CoA dehydrogenase; ACAT, acetyl-CoA acetyltransferase; ACL, ATP citrate lyase; ACC, acetyl-CoA carboxylase; FAS, fatty acid synthase; ELOVL6, long-chain elongase; SCD1, stearoyl-CoA desaturase 1; GPAT, mitochondrial glycerol 3-phosphate acyltransferase; DGAT, diacylglycerol acyltransferase.

The differentially expressed genes were enriched in lipid metabolic process and fatty acid metabolic process (Figure 2), and it is well known that fatty acid metabolism is impaired in diabetic liver [12], [13]. So we analyzed the transcriptional level of enzyme genes participated in fatty acid metabolism. Schematic of three major fatty acid metabolism subprocesses including fatty acid oxidation, fatty acid synthesis and fatty acid storage were shown in Figure 3A. Surprisingly, none of these detected enzyme genes was downregulated in diabetic mouse liver, and most of the enzyme genes in fatty acid oxidation and fatty acid storage were significantly upregulated (Figure 3A–C). For example, *Cpt1a* and *Cpt2* encoding the rate-limiting enzyme carnitine-palmitoyl transferase in fatty acid oxidation were upregulated to 1.36-fold and 1.66-fold respectively in diabetic mouse liver, and *Ehhadh* encoding the key enzyme catalyzing two steps in fatty acid oxidation was increased to 3.99-fold (Figure 3B). Moreover, the enzyme genes *Elovl6*, *Scd1*, *Gpat*, *Dgat1* and *Dgat2*, encoding fatty acid storage enzymes long-chain elongase (ELOVL6), stearoyl-CoA desaturase 1 (SCD1), mitochondrial glycerol 3-phosphate acyltransferase (GPAT) and diacylglycerol acyltransferase (DGAT), were upregulated to 3.01-, 1.89-, 2.37-, 6.67- and 1.48-fold respectively (Figure 3C). Whereas the enzyme genes involved in fatty acid synthesis, including *Acly* and *Fasn* showed no significant change (Figure 3C). These results suggest that fatty acid oxidation and storage are enhanced in 9-week-old diabetic db/db mouse liver.

**Figure 4:**
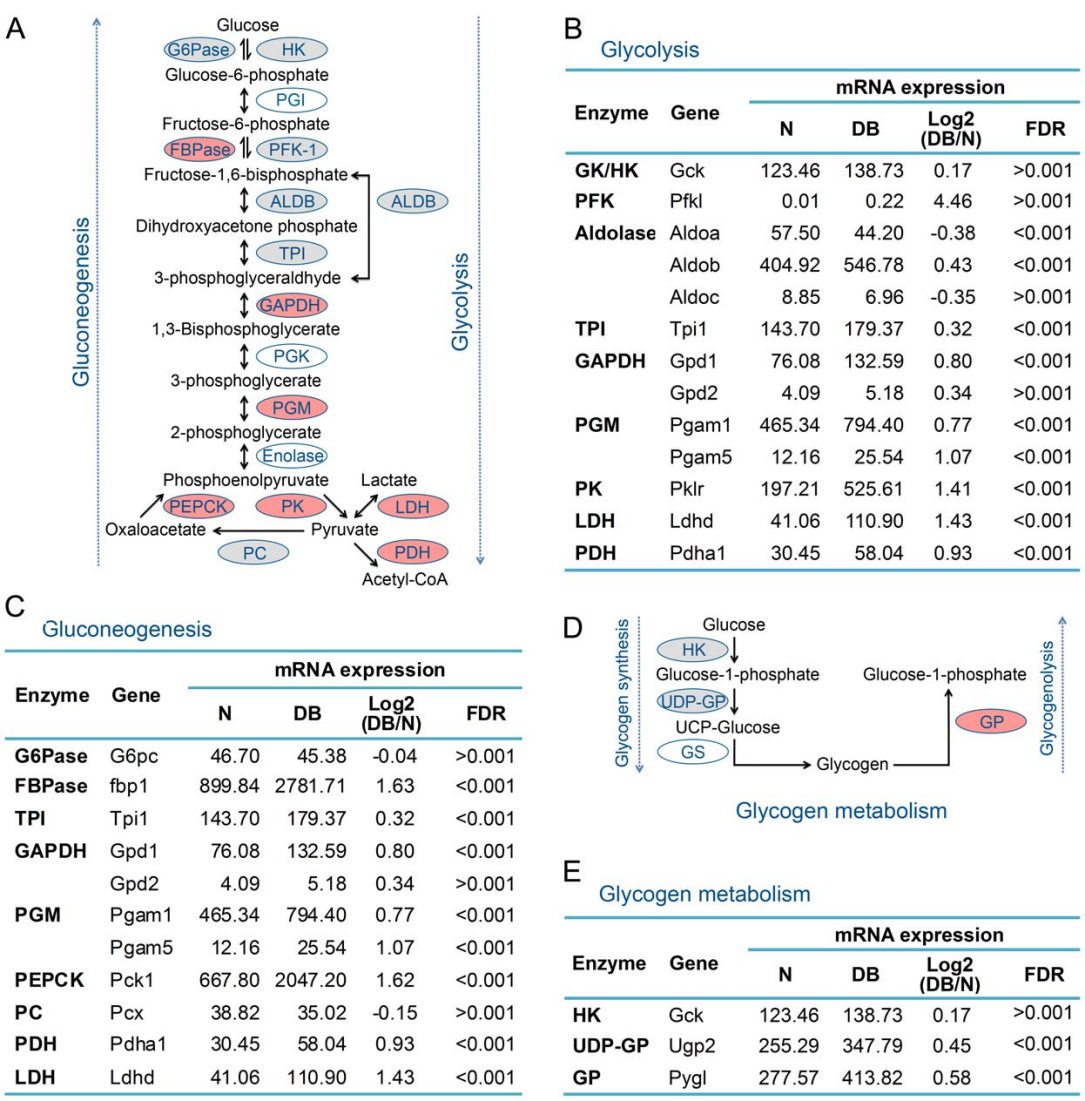
The expression of enzymes directly participated in gluconeogenesis, glycolysis and glycogen metabolism was increased or not significantly changed in diabetic mouse liver. (A, D) Schematic of gluconeogenesis, glycolysis and glycogen metabolism, and the expression of enzymes directly participated in these processes of diabetic mouse liver. The key enzymes include HK, PFK-1 and PK for glycolysis, G6Pase, FBPase and PEPCK for gluconeogenesis, HK, UDP-GP and GS for glycogen synthesis, and GP for glycogenolysis. Red color represents the upregulated genes in diabetic mouse liver with fold change ≥ 1.5 and FDR < 0.001, gray color indicates no significant change, and white color indicates the genes were not detected. (B, C and E) The mRNA levels of the enzymes directly participated in glycolysis, gluconeogenesis and glycogen metabolism in normal (N) and diabetic (DB) mouse liver. HK, hexokinase; PGI, Phosphoglucoisomerase; PFK-1, phosphofructokinase; ALDB, aldolase; TPI, triose phosphate isomerase; GAPDH, Glyceraldehyde 3-phosphate dehydrogenase; PGK, phosphoglycerokinase; PGM, phosphoglyceromutase; PK, pyruvate kinase; LDH, lactate dehydrogenase; PDH, pyruvate dehydrogenase; PC, pyruvate carboxylase; G6Pase, Glucose-6-Phosphatase; FBPase, Fructose 1,6-bisphosphatase; PEPCK, Phosphoenolpyruvate carboxykinase; UDP-GP, Uridine diphosphoglucose pyrophosphorylase; GS, Glycogen Synthase; GP, Glycogen Phosphorylase.

Diabetes is featured with impaired glucose metabolism [4], [5], so we also analyzed the transcriptional level of enzyme genes participated in glucose metabolism. Schematic of four major hepatic glucose metabolism subprocesses including glycolysis, gluconeogenesis, glycogen synthesis and glycogenolysis were shown in Figure 4A and 4D. Surprisingly, none of these detected enzyme genes was downregulated in diabetic mouse liver, and numerous enzyme genes in glycolysis, gluconeogenesis and glycogenolysis were significantly upregulated (Figure 4A–E). For example, *Pklr*, encoding the rate-limiting enzymes pyruvate kinase (PK) in glycolysis [24], was upregulated to 2.66-fold. Meanwhile, both *Fbp1* and *Pck1*, encoding the rate-limiting enzymes FBPase and phosphoenolpyruvate carboxykinase (PEPCK) in gluconeogenesis pathway were upregulated to 3.09- and 3.06-fold respectively. The role of glycogen metabolism is to maintain blood-glucose levels through glycogenesis and glycogenolysis [25]. We found that *Pygl*, encoding the rate-limiting enzyme glycogen phosphorylase in glycogenolysis, was significantly upregulated to 1.49-fold (Figure 4D,E). These results suggest that glycolysis, gluconeogenesis and glycogenolysis are enhanced in 9-week-old diabetic db/db mouse liver.

Taken together, these results suggest that fatty acid and glucose metabolism are markedly enhanced in 9-week-old diabetic db/db mouse liver.

### An overview for the Alteration of Key Genes Involved in Fatty Acid and Glucose Metabolism in Diabetic Mouse Liver

**Figure 5:**
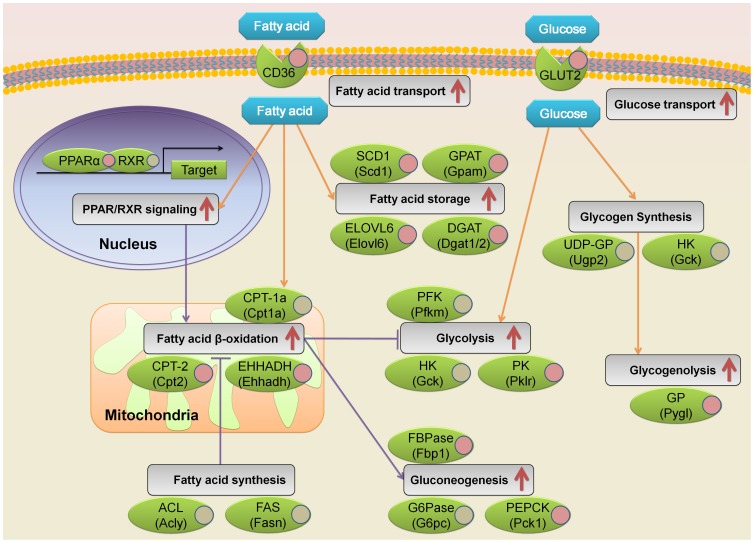
An overview of the key fatty acid and glucose metabolic gene nodes and their alteration in the 9-week-old diabetic db/db mouse liver. The gene nodes and their interactions were obtained from literatures, and the alteration of gene expression was from Table S1. Orange arrows indicate the metabolic flux of glucose and fatty acid. Purple arrows indicate the interaction between different metabolic processes and the indicated signaling pathway. Red arrows show that the indicated process and pathway are expected to be upregulated in diabetic mouse liver based on the detected mRNA levels. Red and gray circles labeled on each gene indicate the genes are upregulated and unaffected in diabetic mouse liver respectively. CD36, fatty acid translocase; GLUT2, glucose transporter 2.

To further elucidate the change of fatty acid and glucose metabolism in diabetic mouse liver, we analyzed more related biological processes and signaling pathways. As shown in Figure 5, besides fatty acid oxidation, synthesis and storage, glycolysis, gluconeogenesis, glycogen synthesis and glycogenolysis, fatty acid and glucose transport and PPAR/RXR signaling were also analyzed and integrated into the schematic of fatty acid and glucose metabolism network in the diabetic mouse liver. Consistent with the expected enhanced fatty acid metabolism in the diabetic mouse liver, we found that the mRNA levels of fatty acid translocase CD36 (*Cd36*) and fatty acid transport protein FATP2 (*Slc27a2*) were increased to 17.7-fold and 1.45-fold respectively (Table S1), suggesting enhanced fatty acid transport in the diabetic mouse liver (Figure 5). The expected enhanced fatty acid transport is consistent with the increased expression of enzyme genes in fatty acid storage (Figure 5) [12], [26]. It is reported that hepatic fatty acid stimulates PPAR/RXR signaling pathway, which can induce transcription of genes responsible for fatty acid β-oxidation [27], [28]. Consistently, we showed that *PPARα* in the diabetic mouse liver was elevated to 2.24-fold accompanied with increased expression of key enzyme genes *Cpt2* and *Ehhadh* in fatty acid β-oxidation, suggesting enhanced fatty acid oxidation in the diabetic mouse liver (Figure 5 and Table S1). Fatty acid oxidation has been reported to stimulate gluconeogenesis and suppress glycolysis [29]. Gluconeogenesis is expected to be upregulated due to the increased expression of the two key enzyme genes *Fbp1* and *Pck1*, however glycolysis is also expected to be increased based on the markedly increased expression of the rate-limiting enzyme gene *Pklr* (Figure 5). The enhanced glycolysis might be associated with the increased glucose transport, which can be expected from the markedly increased the mRNA level of glucose transporter Glut2 (*Slc2a2*) to 3.11-fold (Table S1). In addition, the expression of *Pygl*, encoding the rate-limiting enzyme glycogen phosphorylase in glycogenolysis was significantly upregulated, suggesting increased glycogenolysis (Figure 5). Taken together, Figure 5 provides an overview of the gene nodes involved in fatty acid and glucose metabolism and their alteration in 9-week-old diabetic db/db mouse liver.

### The Potential Relationship of Diabetes with Immunity and Cancer

As shown in Figure 2, the upregulated genes were mainly enriched in metabolic processes, whereas surprisingly the downregulated genes were mainly enriched in immune-related processes. It is well known that inflammation is one of the important factors leads to diabetes [30], [31]. However, the potential relationship of other immunological processes with diabetes is still largely unknown. Here we systematically analyzed the genes in all 15 KEGG pathways in immune system. All the detected genes in the 15 KEGG immunological pathways were shown in Table S2. Notably, for the genes with an average expression level over 5 TPM in normal and diabetic mouse livers, *Asap1* (ArfGAP with SH3 domain, ankyrin repeat and PH domain1), *Cd1d1* (CD1d1 antigen), *Cd36* (CD36 antigen), *Cfd* (complement factor D), *Dntt* (deoxynucleotidyltransferase, terminal), *H2-Q1* (histocompatibility 2, Q region locus 1) and *Zap70* (zeta-chain (TCR) associated protein kinase) in various immunological pathways were increased to over 3-fold, and *Arpc5l* (actin related protein 2/3 complex, subunit 5-like), *C1qa* (complement component 1, q subcomponent, alpha polypeptide), *C1qc* (complement component 1, q subcomponent, C chain), *C6* (complement component 6), *Cldn1* (claudin 1), *Gsn* (gelsolin), *Hspa4* (heat shock protein 4) and *Il1r1* (interleukin 1 receptor, type I) decreased to over 3-fold (Table S2). These data suggest that besides inflammatory signaling, other hepatic immune related pathways are also correlated with type 2 diabetes.

Moreover, according to KEGG analysis in Table 1, the genes downregulated in the diabetic mouse livers were also enriched in pathways in cancer, suggesting that diabetes is correlated with cancer.

**Figure 6:**
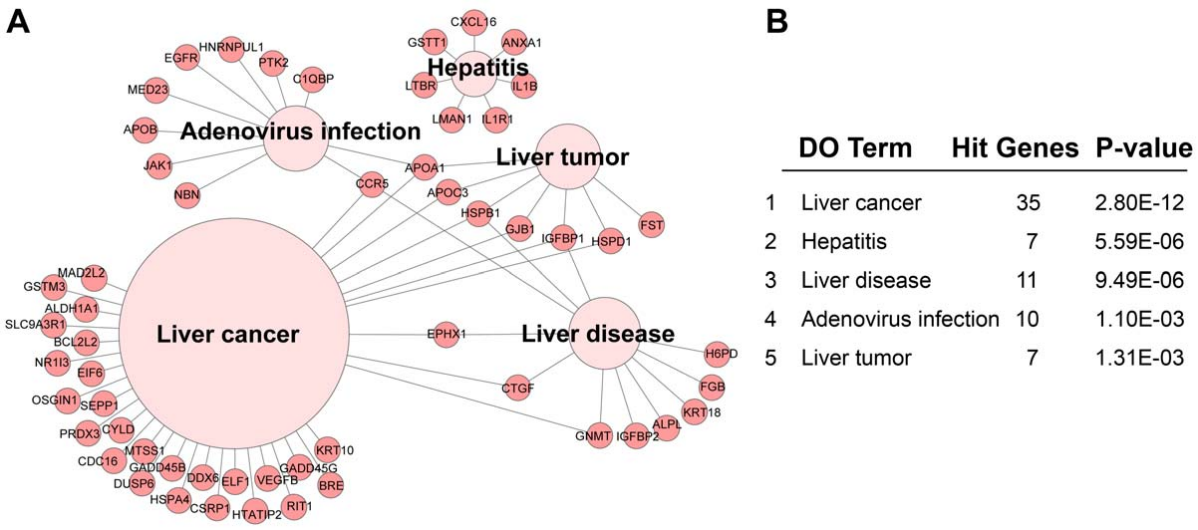
Diabetes is correlated with different liver diseases at a transcriptional view. (A) The map of top 5 liver diseases enriched with the genes altered in 9-week-old db/db mouse liver. 2627 altered genes were assigned to different diseases using the web tool FunDO. The sizes of the disease nodes are proportional to the number of enriched genes. (B) The number of hit genes and *P*-value of the top 5 enriched liver diseases in (A).

To further elucidate the correlations between diabetes and hepatic diseases, we assigned the differentially expressed genes in the diabetic mouse liver to different diseases using web tool FunDO. As shown in Figure 6, 2627 differentially expressed genes were mainly related with liver cancer, hepatitis, liver disease, adenovirus infection and liver tumor. These results are in line with the correlation of diabetes with immunity and cancer [30], [31], [32].

## Discussion

This study provides the gene expression profile data of type 2 diabetic mouse liver by high-throughput sequencing, and demonstrates the main biological processes, pathways, fatty acid and glucose metabolism related key enzyme genes and liver diseases correlated with type 2 diabetes. Type 2 diabetes is mainly correlated with the hepatic metabolic processes, especially fatty acid and glucose metabolism, and is also correlated cancer and immune-related liver diseases. These results should be helpful for further understanding the metabolic changes in type 2 diabetic liver and the correlation of diabetes with hepatic cancer and immunity.

High-throughput RNA sequencing approach to measure gene expression levels greatly increases our ability to quantitatively detect mRNA level in an relatively unbiased way [22]. With this technology, we showed the top 15 abundance change genes upregulated or downregulated, and the top 15 fold change genes increased or decreased in Figure 1. To our knowledge, there is no published paper comparing the hepatic gene expression profile of wild type and db/db mice using microarray data, so we briefly compared our data with the reported microarray data from 10-week-old diabetic ob/ob mice [33]. Among the top 15 abundance change genes upregulated or downregulated, almost all these genes increased or decreased similarly, except *Pck1* encoding PEPCK, a key enzyme in gluconeogenesis. It has been reported that *Pck1* mRNA level was increased in diabetic liver [20], [34], which is in line with our data. Moreover, we found that some genes such as *Comt1*, *Osgin1*, *Mup-ps13* and *Gm4738* at a relatively high abundance were quantified in this study but not found in the previous microarray data [33]. Interestingly, we also found a few largely unknown and markedly differentially expressed genes including *Mup-ps13* and *Gm4738*, and the role of these genes in liver is yet to be elucidated. Similar with the top abundance change genes, the change trend of top 15 fold change genes in our observation is the same as previous microarray data [33]. Among the top 15 fold change genes upregulated or downregulated by diabetes, 15 genes were clustered in metabolic process, such as *Cyp2b9*, *Sult1e1*, *Hsd3b5* and *Cyp2b13*. Among the other 15 genes not in metabolic process, *Vdac2* has been reported to inhibit mitochondrial apoptosis [35], and *A1bg* is specifically expressed in liver as shown in BioGPS (http:// biogps.gnf.org). However, the role of *Vdac2* and *A1bg* in glucose and lipid metabolism is yet to be elucidated. Future studies focused on these markedly differentially expressed hepatic genes with largely unknown effects on glucose and lipid metabolism will provide new insights into the molecular basis of type 2 diabetes.

The role of hepatic fatty acid oxidation in diabetes is complicate. Metformin, a widely used drug for type 2 diabetes, activates AMPK, induces fatty acid oxidation and inhibits lipogenesis [36]. The increased fatty acid oxidation in mice with *Acc2* deficiency prevented the onset of type 2 diabetes [37]. However, fatty acid oxidation inhibitors have been considered to treat type 2 diabetes [38]. It has been reported that increased hepatic fatty acid oxidation can enhance gluconeogenesis, and thus stimulates hepatic glucose production [38]. In this study, we found that most of the hepatic enzyme genes in fatty acid oxidation were significantly upregulated (Figure 3A,B), which is consistent with the increased expression of the gluconeogenesis enzyme genes (Figure 4A,C). The dual role of hepatic fatty acid oxidation might be based on the balance of reducing free fatty acid level and stimulating hepatic glucose production by enhancing gluconeogenesis. In addition, consistent with previous reports [39], [40], the fatty acid storage enzyme genes *Elovl6*, *Scd1*, *Gpat*, *Dgat1* and *Dgat2* were markedly upregulated. Taken together, our results suggest that fatty acid metabolism is markedly enhanced in 9-week-old diabetic db/db mouse liver, implicating the diabetic mouse liver is working hard on fatty acid homeostasis and trying to solve the problem of impaired fatty acid and glucose metabolism.

It has been reported that the rate of hepatic gluconeogenesis in diabetes is elevated, [41], [42], [43]. Consistent with the previous reports, here we also found that the mRNA levels of rate-limiting enzymes FBPase and PEPCK in gluconeogenesis pathway were upregulated (Figure 4C). In glycolysis pathway, it is reported that the pyruvate kinase, glucokinase and phosphofructokinase (PFK) enzyme activities and the pyruvate kinase and glucokinase mRNA levels are decreased in diabetic liver [41]. Interestingly, in this study we found that the mRNA levels of glucokinase and phosphofructokinase were not significantly altered in diabetic liver, and the mRNA level of pyruvate kinase even increased (Figure 4B). The decrease of phosphofructokinase activity in diabetic rat liver is resulted from accelerated protein degradation rate while the synthetic rate remains nearly normal [44], which is consistent with no significant change of phosphofructokinase mRNA level in diabetic mouse liver in our study. The increased mRNA level of pyruvate kinase and unchanged mRNA level of glucokinase observed in this study implicate that the liver of 9-week-old db/db mice at a relative early stage of diabetes might be able to enhance glycolysis to reduce glucose level [45], [46], [47]. About 90% increase of hepatic glucose production in type 2 diabetic subjects is accounted by accelerated gluconeogenesis [42]. The remaining about 10% increase of hepatic glucose production in type 2 diabetic subjects is likely mainly contributed by slightly increased glycogenolysis [42], which is consistent with nearly 50% mRNA-level increase of glycogen phosphorylase (Figure 4E), the key enzyme of glycogenolysis [48]. Taken together, our data implicate that although glycolysis was enhanced to consume more glucose, gluconeogenesis and glycogenolysis were also accelerated to produce more glucose (Figure 5). Thus the 9-week-old diabetic db/db mouse liver had limited effect to improve blood glucose levels, although it showed accelerated glucose metabolism.

Inflammation is considered as one of the important factors causing the development of type 2 diabetes [49], [50], [51]. Similarly, we also found many immune-related genes were markedly changed in type 2 diabetic mouse liver (Table S2). To our surprise, genes downregulated in diabetic mouse liver were mainly enriched in immune-related processes, such as adaptive immune response and lymphocyte mediated immunity (Figure 2B). These findings suggest that type 2 diabetes is associated with altered hepatic immune response. Consistently, it is reported that the frequency of hepatitis B and C virus infection is increased in type 2 diabetic patients [52], [53], [54], which is also in line with the association of hepatitis with type 2 diabetes (Figure 6). These findings suggest that the alteration of immune-related genes observed in this study may facilitate the infection of hepatitis B and C virus. Moreover, it has been reported that the liver has immune-related functions, such as removal of pathogens and antigens, which requires a local immune response [55], [56]. Sinusoidal endothelial cells and Kupffer cells in liver can directly interact with passenger leukocytes, and should be involved in the local immune response. Selective disruption of hepatic resident macrophages, Kupffer cells, has been shown to be sufficient to improve hepatic insulin sensitivity in high-fat diet model [57], [58]. These previous findings suggest that the downregulated immune-related genes observed in our study might play important roles in Kupffer cells or other hepatic immune response cells in response to the development of diabetes. Future studies focused on these downregulated immune-related genes such as in Kupffer cells might provide new insights for the development of hepatic insulin resistance and type 2 diabetes.

Epidemiological studies clearly indicate that the risk of several types of cancer including liver cancer, is increased in diabetic patients [59], [60], [61]. The possible biologic links between diabetes and cancer risk include hyperinsulinemia, hyperglycemia or chronic inflammation, but the underlying molecular mechanisms are still largely unknown [59]. In this study, analysis of the hepatic gene expression pattern by Fundo also suggests that type 2 diabetes is highly associated with liver cancer (Figure 6). Moreover, our study provides the genes affected by type 2 diabetes and associated with liver cancer (Figure 6). Future studies focused on these candidate genes might shed new light on the molecular mechanisms for the high risk of liver cancer in diabetic patients.

Taken together, this study shows the quantitative gene expression profile and the alteration of biological processes, pathways, and key enzyme genes in fatty acid and glucose metabolism in type 2 diabetic mouse liver. Moreover, we found that hepatic local immune response and liver cancer are associated with type 2 diabetes. These findings provide new insights for the alteration of hepatic fatty acid and glucose metabolism and involvement of hepatic immunity in the development of type 2 diabetes.

## Materials and Methods

### Animal Experiments

All animal experimental procedures were approved by the Institutional Animal Care and Use Committee of the Institute for Nutritional Sciences (Protocol number 2007-AN-9). C57BL/6 male mice and diabetic db/db male mice at the age of 7 weeks obtained from SLAC (Shanghai, China) and Model Animal Research Center of Nanjing University (Nanjing, China) were divided into two groups for 12 mice per group, and were allowed to have access to water and chow diets *ad libitum*. About 3 h after the beginning of light cycle, 9-week-old mice were sacrificed, and the livers were immediately collected and snap-frozen in liquid nitrogen.

### Sample Preparation and Solexa Library Construction

Liver samples were ground in liquid nitrogen, and total RNA was extracted with Trizol reagent (Invitrogen). The integrity of total RNA was evaluated using an Agilent Bioanalyzer 2100. Solexa libraries were constructed following the manufacturer’s standard according to the schematic as described previously [62].

### Solexa Sequencing and Data Analysis

The image files obtained from Illumina 1G sequencer were processed to produce sequence data. Then the high-quality reads were screened from the original raw data, and the adaptors were removed from each sequence. Subsequently, high quality clean tags were compared with RefSeq database and the expression level of each gene was normalized to transcripts per million (TPM). The raw data and the processed gene expression data were deposited in GEO with a series number GSE43314, including GSM1060446 and GSM1060447 for normal and diabetic mouse liver respectively. The significance of digital gene expression profiles was analyzed as described previously [63]. The differentially expressed genes were classified into upregulated and downregulated groups as Cluster A and B respectively, and then the expression pattern of these genes was visualized using the heat-map function in the R base package [64].

### Biological Process and Pathway Analysis

The clustered genes were assigned to biological processes based on Gene Ontology using the web tool DAVID (http://david.abcc.ncifcrf.gov/home.jsp) [65], [66]. Hypergeometric test was used to select the enriched biological process in Gene Ontology for each cluster. The pathways associated with these gene clusters were analyzed by KEGG (www.genome.jp/kegg/pathway.html) [67].

### FunDo Analysis

To study the correlation of disease and gene expression change, the differentially expressed genes in db/db mouse liver were assigned to different diseases based on Disease Ontology and peer-reviewed evidence from GeneRIF using the web tool FunDO (http://django.nubic.northwestern.edu/fundo/) [68]. Then the gene-disease interaction networks were visualized by Cytoscape v2.6.2 [69].

## Supporting Information

Figure S1 **Genes differentially expressed in normal and diabetic mouse liver.** (A) 8551 genes with average TPM no less than 1 in normal and diabetic mouse liver were selected to analyze the gene expression profile. Number of genes differentially expressed in diabetic mouse liver compared with normal control according to the indicated fold change and FDR value was listed. 2627 genes with absolute fold change ≥ 1.5 and FDR < 0.001 were considered as differentially expressed genes in this study. (B) Heat-map images for the 2627 differentially expressed genes. The selected genes were classified into Cluster A or B, based on the genes upregulated or downregulated in diabetic mouse liver. Red and blue indicate genes with high and low abundance respectively.
(TIF)

Table S1 **Gene expression data of normal and diabetic mouse liver.** The 8551 genes with average TPM no less than 1 in normal and diabetic mouse liver were listed. N_TPM, the TPM of the indicated genes in normal mouse liver; DB_TPM, the TPM of the indicated genes in diabetic mouse liver.
(XLS)

Table S2 **Gene expression profile of immune-related 15 pathways in KEGG database in diabetic (DB) mouse liver compared with normal (N) control.**
(XLS)
