# Supplementary figures and images for: Gene Expression Profile Analysis of Type 2 Diabetic Mouse Liver
Source: PLoS One. 2013 Mar 1;8(3):e57766. doi: 10.1371/journal.pone.0057766 (PMC3585940; doi:10.1371/journal.pone.0057766)

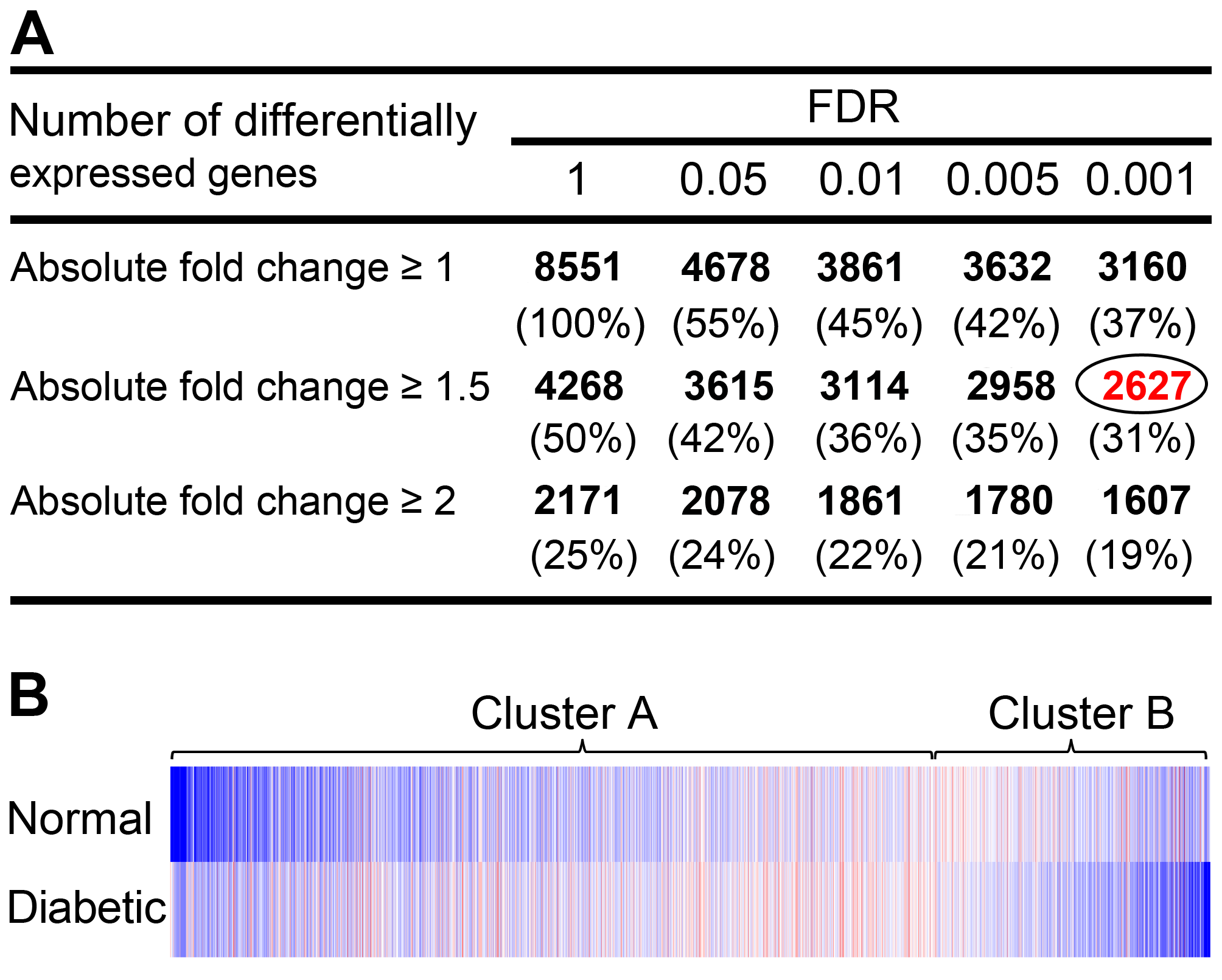

Supplement: Figure S1 — Genes differentially expressed in normal and diabetic mouse liver. (A) 8551 genes with average TPM no less than 1 in normal and diabetic mouse liver were selected to analyze the gene expression profile. Number of genes differentially expressed in diabetic mouse liver compared with normal control according to the indicated fold change and FDR value was listed. 2627 genes with absolute fold change ≥ 1.5 and FDR < 0.001 were considered as differentially expressed genes in this study. (B) Heat-map images for the 2627 differentially expressed genes. The selected genes were classified into Cluster A or B, based on the genes upregulated or downregulated in diabetic mouse liver. Red and blue indicate genes with high and low abundance respectively. (TIF) [file pone.0057766.s001.tif]
